# Subjective achievement from psychiatry rotation in the Japanese postgraduate residency system: a longitudinal questionnaire study

**DOI:** 10.1186/s12909-022-03712-0

**Published:** 2022-08-27

**Authors:** Yusuke Matsuzaka, Koichi Taniho, Kengo Maeda, Shintaro Sakai, Toru Michitsuji, Eriko Ozono, Yoshiro Morimoto, Hirohisa Kinoshita, Kayoko Matsushima, Hisayuki Hamada, Akira Imamura, Hirokazu Kumazaki, Hiroki Ozawa

**Affiliations:** 1 Department of Neuropsychiatry, Nagasaki University Graduate School of Biomedical Sciences, 1-7-1 Sakamoto, Nagasaki city, 852-8501 Japan; 2 Medical Education Development Center, Nagasaki University Hospital, Nagasaki city, Japan; 3 Health Center, Nagasaki University, Nagasaki city, Japan; 4 Department of Occupational Therapy Sciences, Nagasaki University Graduate School of Biomedical Sciences, Nagasaki city, Japan; 5 Department of Future Mental Health Sciences, Nagasaki University Graduate School of Biomedical Sciences, Nagasaki city, Japan

**Keywords:** Competency items, Confidence in initiating treatment, Postgraduate residency system, Psychiatry rotation, Subjective understanding

## Abstract

**Background:**

Psychiatry rotation has been mandatory in the Japanese postgraduate residency system since 2020. Some psychiatry-related competency items are stipulated as mandatory for residents. The current study aimed to clarify whether psychiatry rotation affected residents’ subjective achievement of these competency items.

**Methods:**

This longitudinal study was conducted among postgraduate residents who completed a rotation in the psychiatry department at Nagasaki University Hospital across two academic years (2020–2021). The survey was administered at the start and at the end of the psychiatry rotation. Residents evaluated their subjective understanding and confidence regarding initiating treatment for these competency items using a six-point Likert scale. The average scores for each item were compared between pre-rotation and post-rotation.

**Results:**

In total, 99 residents (91.7%) responded to this survey. Residents had significantly higher scores at post-rotation compared with pre-rotation in all psychiatry-related competency items in both subjective understanding and confidence in initiating treatment. Additionally, strong effect sizes were found for many items.

**Conclusion:**

Residents improved learning about psychiatry-related competency items through psychiatry rotation. This finding suggests that it is reasonable for psychiatry rotation to be mandatory in the current Japanese postgraduate residency system. The importance of psychiatry is likely to increase in both undergraduate and postgraduate medical education in the future. It is necessary to continuously update educational strategies to meet changing social needs over time. As this study was conducted at a single institution, a multi-center study is needed to expand the current findings.

## Background

Training programs for doctors in Japan have three main phases: 6 years of general training in medical school, including 2 years of clinical clerkship; 2 years of general training as a postgraduate resident; and approximately 3–5 years of training in each chosen specialty. Medical students and residents need to complete their training in primary care including psychiatric disorders commonly encountered in general medical practice [1]. The current Japanese postgraduate residency system was started in 2004 by the Japanese Ministry of Health, Labour and Welfare. The purpose of this system was to improve residents’ treatment skills in primary care [2]. Residents do not have any specialty during the 2 years. This curriculum initially included a psychiatry rotation of more than 1 month to ensure that all doctors achieved a certain level of proficiency in assessing psychiatric disorders [3]. However, this curriculum was relaxed in 2010 to prepare residents for various post-residency careers, and the psychiatry rotation became elective. Residents reported less confidence regarding clinical competency items under the relaxed curriculum, including in the diagnosis of depression [4]. In 2020, the psychiatry rotation again became mandatory following further revision of the residency system, which reflected the increased need for all doctors to have a general ability to treat psychiatric symptoms and diseases.

The teaching guidelines of the Japanese residency system stipulate certain competency items regarding symptoms and diseases, which residents must experience and appropriately respond to. These items are often encountered in general practice. Some psychiatric items are also included, and new items are added in the revision to current system (e.g., dependence, agitation and delirium, and disorders of growth and development). Residents are expected to make appropriate initial assessment and medication for these items after completing rotation in psychiatry. This reflects the increasing need to respond to combinations of physical and mental illness [5–7]. Residents do not have to take questionnaire tests or objective structured clinical examinations [8] at the end of rotation, because of the shortage of clinical teachers.

In addition to objective assessment by clinical teachers, learners’ self-assessment is also important [9]. In the previous system, residents who did not complete the psychiatry rotation showed a lower level of subjective understanding and confidence in initiating treatment toward psychiatry-related competency items [10]. In the current system, all residents must undertake rotation in psychiatry, including those who are not interested in psychiatry, and those who are strongly interested in another career. Given this situation, it is necessary to assess the educational effects of mandatory psychiatry rotation. Impact of psychiatry rotation often has been discussed from the point of attitudes toward psychiatry, stigma towards mental illness, and psychiatry as a career choice [11]. There are few studies assessing educational effect of psychiatry rotation from the point of competency required in postgraduate residents. The current study aimed to clarify whether psychiatry rotation affected residents’ subjective achievement of competency items related to psychiatry in the current Japanese residency system.

## Methods

At Nagasaki University Hospital, most residents complete a 1-month rotation in the psychiatry department. Approximately 4–5 residents simultaneously undertake rotation in the psychiatry department per term. Residents participate in patient treatment with staff psychiatrists in the psychiatric ward, psychiatric outpatient clinic, and other department wards as a consultation-liaison activity. There are 5–6 clinical teachers in the psychiatry department, who check and approve residents’ treatment activity.

The current longitudinal study was conducted among postgraduate residents who undertook rotation in the psychiatry department at Nagasaki University Hospital across two academic years (2020–2021). 108 residents were invited to participate. The survey was administered both at the start and at the end of psychiatry rotation. We selected nine psychiatry-related competency items (dependence, mood disorders, agitation and delirium, disorders of growth and development, schizophrenia, dementia, insomnia, memory deficit, and depression) from the guidelines of the Japanese residency system (up to 2019 and after 2020), in accord with a previous study [10]. Mood disorders and insomnia were selected from the pre-2019 guidelines, and the other items were selected from the guidelines after 2020. Residents were asked to evaluate their subjective understanding and confidence in initiating treatment for these competency items using a six-point Likert scale (1: not understand at all / not confident at all – 6: completely understand / completely confident). Participants were informed about this survey via a written leaflet and invited to complete the questionnaire. Participation was voluntary and uncompensated, and potential participants were informed that there was no penalty for non-participation. Responses to the questionnaire were anonymous. Participants’ consent was assumed if participants responded. Paired t-tests were used to compare the average scores for each item between pre-rotation and post-rotation. We considered *p*-values < 0.006 to indicate statistical significance using the Bonferroni correction. The statistical analyses were performed with IBM SPSS Statistics version 27.0 for Windows (IBM, Tokyo, Japan). This study was approved by the Ethical Review Board of Nagasaki University.

## Results

In total, 99 residents (91.7%) responded to this survey. Table 1 shows participants’ characteristics, 33 (31.4%) of whom were female residents, and 66 (62.9%) of whom were male. In addition, 65 (61.9%) residents belonged to the university hospital, and 34 (32.4%) were at a community-based hospital. Table 2 shows the average scores for subjective understanding and confidence in initiating treatment. Residents had significantly higher scores for subjective understanding at post-rotation than at pre-rotation in all nine competency items. Similarly, participants had significantly higher scores for confidence in initiating treatment at post-rotation than at pre-rotation for all nine competency items.

**Table 1:** Participants’ characteristics

Number of residents	108
Number of participants	99
Response rate (%)	91.7
Age (average)	27.1
Standard Deviation	2.75
Gender	
Male	66 (62.9%)
Female	33 (31.4%)
Curriculum type	
University hospital	65 (61.9%)
Community-based hospital	34 (32.4%

**Table 2:** Residents’ subjective understanding and confidence in initiating treatment of competency items related to psychiatry

psychiatry-related competency items	Subjective understanding				Confidence in initiating treatment			
	Pre-rotation	Post-rotation	*p*-value	Cohen’s d	Pre-rotation	Post-rotation	*p*-value	Cohen’s d
Dependence	2.35	2.89	< 0.001**	0.54	1.84	2.45	< 0.001**	0.66
Mood disorder	2.55	3.36	< 0.001**	0.88	2.01	2.89	< 0.001**	0.95
Agitation and delirium	2.61	3.74	< 0.001**	1.21	2.07	3.37	< 0.001**	1.33
Disorder of growth and development	2.14	2.54	< 0.001**	0.40	1.70	2.12	< 0.001**	0.45
Schizophrenia	2.46	3.31	< 0.001**	1.07	1.68	2.73	< 0.001**	1.14
Dementia	2.92	3.43	< 0.001**	1.05	2.37	3.02	< 0.001**	0.64
Insomnia	2.78	3.76	< 0.001**	1.06	2.41	3.48	< 0.001**	0.95
Memory deficit	2.68	3.27	< 0.001**	0.96	2.20	2.91	< 0.001**	0.80
Depression	2.52	3.40	< 0.001**	0.90	1.95	2.98	< 0.001**	1.20

6-point Likert scale from 1 (low) to 6 (high)

^**^: *p* < 0.01

## Discussion

Residents had higher scores on all nine competency items at post-rotation. Additionally, large effect sizes were seen for many items. Residents appeared to improve their understanding and confidence in initial treatment through psychiatry rotation. Mandatory psychiatry rotation is reasonable and worthwhile, and may constitute an essential and effective part of the residency curriculum. However, it is unclear whether these post-rotation scores are suitable for the residents completing psychiatry rotation. It is also unclear what the one-point increasing of the scale means. The post-rotation score of confidence in initial treatment seemed to be low in most items. Postgraduate residents are expected to be ready to practice across the full breadth of their chosen field [12]. They are often assessed their performance (i.e., “Shows how”) and action (i.e., “Does”) [13] in the workplace, so called entrustable professional activity [14–16]. Increasing the post-rotation score of confidence in initial treatment are needed by continuous improvement of educational program in the future. Basic psychiatric knowledge and treatment skills are useful in various fields, and even some specialty fields require a psychiatric training program [17–19]. The need for psychiatric knowledge and skills is expected to continue to grow in the future. The importance of psychiatry is also likely to increase in both undergraduate and postgraduate medical education. It is necessary to repeatedly update educational strategies to meet changing social needs.

A previous study reported that psychiatry rotation had no significant educational effects on knowledge about disorders of growth and development [10]. However, in the current study, residents achieved sufficient learning on this item. This item reflects a broad concept that can be experienced in both psychiatry (neurodevelopmental disorders and truancy) and pediatrics (failure to thrive). It was previously assumed that residents may not clearly recognize this item as a psychiatric competency. Child and adolescent mental health have recently been recognized as leading problems among primary care physicians, and their importance is increasing [20, 21]. In the Japanese residency system, all residents are expected to commit to working in child and adolescent mental health during the 2 years curriculum. Currently, residents may devote more study to psychiatric concepts related to this area, such as neurodevelopmental disorders, compared with residents several years ago.

Although the current study focused on the competency items, the residency system also contains other various objectives (e.g., communication skills, ethics, and interprofessional collaboration). Some of these objectives may be expected to be learned deeply during psychiatry rotation. There is no detailed description regarding how and what must be learned during psychiatry rotation in the teaching guidelines of the Japanese residency system. Clinical teachers in psychiatry can provide model attitudes and relationships, as well as knowledge and skills [22]. It would be useful to clarify what residents should achieve in a more multifaceted way through psychiatry rotation. Furthermore, psychiatrists are needed as educators in various areas of medical education not only in psychiatry rotation [23].

The present study involved several limitations. First, the study was conducted at a single institution. Various strategies are implemented in clinical training during psychiatry rotation. Thus, it would be preferable to conduct future studies at multiple institutions. Second, the questionnaire used in this study was self-designed by the authors, and its validity and reliability may therefore be insufficient. Because few previous studies evaluated residents’ subjective achievements in the field of psychiatry, a self-designed questionnaire was necessary in this study. Third, social desirability bias may have affected the current results, if residents answered in a manner that they felt would be viewed favorably by others.

Psychiatry is only a part of various specialty in medicine. However, many doctors other than psychiatrist are expected to deal with psychiatric contents. Despite ongoing discussions about what should be taught during the student and residency periods, widely accepted conclusions have not yet been reached. There are substantial differences in educational content related to psychiatry between medical schools and postgraduate training curricula in various countries [24–26]. Further research is needed to expand the current findings.

## Conclusion

Residents were found to have improved their understanding and confidence in initial treatment through psychiatry rotation in terms of psychiatry-related competency items. Mandatory psychiatry rotation is reasonable and worthwhile in the current Japanese postgraduate residency system. The importance of psychiatry is likely to increase in both undergraduate and postgraduate medical education in the future. It is necessary to continuously update educational strategies to meet changing social needs over time.

## Data Availability

All data generated or analysed during this study are included in this published article.
